# Fertility preservation decision making amongst Australian transgender and non-binary adults

**DOI:** 10.1186/s12978-018-0627-z

**Published:** 2018-10-25

**Authors:** Damien W. Riggs, Clare Bartholomaeus

**Affiliations:** College of Education, Psychology and Social Work, Flinders University, GPO Box 2100, Adelaide, SA 5001 Australia

**Keywords:** Transgender, Non-binary gender, Decision making, Fertility preservation, Reproductive desires

## Abstract

**Background:**

Historically, transitioning gender was seen as precluding transgender people from having children in the future. However, there are now increased reproductive options available to transgender people, with such options also available to non-binary people (i.e., people whose gender is not exclusively male or female). These options include undertaking fertility preservation if genetic children may be desired in the future. Despite these increased options, there is still only a limited amount of international research exploring the views of transgender and non-binary people on fertility preservation.

**Methods:**

This mixed-methods study draws on a convenience sample of Australian transgender and non-binary adults, focused on their decision making about fertility preservation. The questionnaire was constructed by the authors, drawing on previous research. Participants were recruited via Australian organisations and groups made up of and/or working with people who are transgender or non-binary. The questionnaire was open from January–February 2018. The final sample included 409 participants. Statistical analyses were conducted on the closed-ended responses. Open-ended responses were analysed via a conventional content analysis.

**Results:**

Decisions about fertility preservation were influenced by views on the importance of genetic relatedness, willingness to delay transition, economic resources, already having children or desiring children in the future, and the views of significant others. Advice or counselling prior to decision making was received only by a minority of participants. Very few participants (7%) had undertaken fertility preservation, although 95% said that fertility preservation should be offered to all transgender and non-binary people. Participants who viewed genetic relatedness as important were more likely to have undertaken fertility preservation.

**Conclusions:**

The findings indicate that fertility preservation should be made available as an option to all transgender or non-binary people prior to undertaking treatment which may impact on fertility. However, it should also be recognised that not all people who are transgender or non-binary will want to undertake fertility preservation, and that not all people may be able to afford to.

## Plain English summary

In the past, transgender people were seen as automatically excluded from having children. However, the reproductive rights of transgender people have been increasingly recognised, as too have those of non-binary people (i.e., people whose gender is not exclusively male or female). This may include preserving fertility should transgender or non-binary people wish to have children in the future. Yet there is little research which explores the views of transgender and non-binary people on fertility preservation.

To explore such views, a questionnaire was designed by the authors. The questionnaire was completed by 409 transgender and non-binary adults in Australia.

The findings from the questionnaire show that the people who completed the survey made decisions about fertility preservation depending on several reasons. The reasons included how important it was to be genetically related to a child, how willing they were to wait to transition gender, how much money they had, whether they had children already or if they wanted to have children in the future, and the views of people around them. Only a small number of people who completed the survey had preserved their fertility. Nearly all said that fertility preservation should be offered to all transgender and non-binary people.

The findings suggest that the option for preserving fertility should be available to all people who are transgender or non-binary. However, not all transgender or non-binary people will want to preserve their fertility, or will be able to afford it.

## Background

Historically, transitioning gender was seen as precluding transgender people from having children in the future, with sterilisation often being mandatory, or treated as axiomatic in the context of gender affirming surgery. As such, whilst some transgender people may have had children prior to transitioning, the potential for future children was typically not considered. The World Professional Association for Transgender Health (WPATH) *Standards of Care for the Health of Transsexual, Transgender, and Gender Nonconforming People* (SOC), however, in their seventh version [1], introduced a focus on reproductive rights. The SOC recommend that fertility preservation should be offered to anyone considering undertaking medical treatment which may have a permanent impact on their fertility. Such treatment specifically relates to gender affirming surgeries undertaken by transgender women or men which lead to irreversible sterility, and hormone therapies that such women and men may undertake that can permanently impact on gamete quality [2]. People with a non-binary gender (i.e., people whose gender is not exclusively male or female) may undertake similar medical treatments, although research suggests that non-binary people may be less likely to do so than transgender people who have a binary gender. [3] Importantly, however, such research also suggests that the lower rates at which non-binary people access medical treatments may be a product of perceived or actual barriers to services arising from a lack of clinician awareness about non-binary genders. [3] Perhaps as a consequence of such a lack of awareness, the experiences of non-binary people in terms of reproduction have often been overlooked in previous research, or as will be seen below, are often collapsed in with the experiences of transgender people who have a binary gender. This is a problem given non-binary people are likely to have specific needs and experiences.

The country in which transgender or non-binary people live constitutes a formative context in terms of experiences of fertility preservation. Therefore, it is important to provide a brief background to the Australian context as it is the setting for the study reported in this paper. There are no specific laws or policies banning transgender or non-binary people from undertaking fertility preservation or assisted reproductive technology in Australia, although in some jurisdictions legislation enables providers to object on religious grounds. However, fertility preservation is expensive and is only covered by the Australian public healthcare system if it is classified as being medically necessary. Whilst for oncology patients fertility preservation is seen as medically necessary, for transgender and non-binary people it is not. Estimates from 2010 suggest that, depending on the clinic, sperm banking costs $300–$400 a year in storage fees, sperm aspiration costs $1000–$1500, egg banking or embryo storage costs $300–$500 a year in storage fees, and IVF may cost $15,000–$20,000 (all in Australian dollars) [4]. These costs must be located in the context of legislation, which in some Australian states mandates that in order for a person to change their gender marker on their birth certificate, they must have undergone some form of surgery, which for many people will result in sterility. As such, not only is fertility preservation costly in Australia, but for many transgender and non-binary people it is also necessary if they wish to preserve their fertility prior to undertaking surgeries that will allow for formal recognition of their gender.

In terms of the importance of fertility preservation, previous research suggests that whilst lower numbers of transgender (and in some research non-binary) people are already parents than would be expected from general population data, the numbers of transgender and non-binary people who wish to become parents in the future is not negligible [5–7]. These studies report that transgender and non-binary people become parents in multiple ways, including by giving birth, by a partner giving birth, by surrogacy, by foster care or adoption, or by step parenting. As the latter pathways to parenthood would suggest, existing research indicates that transgender or non-binary people may be less focused on being genetically-related to their children than the broader population [7–10]. Studies have also found that transgender women are more likely to already have children than transgender men [7, 11, 12].

Whilst there are increasing options available for transgender or non-binary people to undertake fertility preservation, there is still only a limited amount of international research exploring the views of transgender people on this topic, and even less with non-binary people. De Sutter et al.’s [11] groundbreaking study examined the views of 121 transgender women, most of whom lived in Europe. Their study found that over three quarters of respondents thought that sperm freezing should be offered to all transgender women before hormonal treatment, although only half would have seriously thought about doing this or would have done it themselves if it had been available. A similar study conducted more recently with 50 transgender men in Belgium found that 37.5% of respondents would have considered freezing germ cells if the technique had been available [12]. Similarly, literature drawing on case reviews highlights that the uptake of fertility preservation is low [13], particularly amongst transgender young people [14, 15]. Research has also found that to date few people have used their preserved gametes to conceive children [14]. In terms of gender, previous research and case reviews indicate that transgender women are more likely to undertake fertility preservation than transgender men [8, 14, 16].

In terms of decision making about fertility preservation, studies have found that this can be influenced by perceived psychological and physiological challenges associated with either ceasing hormone treatments in order to undergo fertility preservation [17], or delaying surgical and medical transition [8, 11, 17]. Decision making about fertility preservation may also be influenced by the associated financial costs [8, 9, 18], along with not being aware that fertility preservation is an option, not knowing that hormone treatments may make it difficult to have genetically-related children, travel time to the nearest fertility clinic, and the newness of technologies such as egg freezing [8, 19]. In terms of individual factors, research has found that for some people the idea of using stored gametes to conceive a child can be dysphoria inducing, and that some individuals express concerns about passing on poor genes [11].

Whilst previous research has included comment on decision making, as summarised above, this has occurred in the context of research on intentions, experiences and outcomes for transgender (and occasionally non-binary) people who plan to undertake, or who have already undertaken, fertility preservation. The present study sought to focus specifically on decision making about fertility preservation amongst a convenience sample of transgender and non-binary people, and was guided by the following research questions:

1. Is genetic relatedness valued, does this relate to undertaking fertility preservation, and how is genetic relatedness accounted for?
2. How does gender-affirming medical treatment relate to fertility?
3. How prevalent is fertility preservation and who is most likely to undertake it?
4. What influences decision making about fertility preservation, and does decision making occur in the context of professional advice or counselling?

## Methods

The mixed-methods study design was intended as a scoping study given the relative lack of previous research specifically on decision-making. Ethical approval for the research was granted by the Flinders University Social and Behavioural Research Ethics Committee (project number 7867).

### Participant recruitment

Participants were recruited via emails to Australian organisations and groups made up of and/or working with people who are transgender or non-binary, including broader ‘LGBT’ organisations. Organisations and groups were asked to share information about the questionnaire via their social media (Facebook and/or Twitter) and via an email to their members. Thirty-five organisations and groups were contacted and approximately half shared the details of the questionnaire. The researchers did not have access to member names or email addresses. The questionnaire was open from January–February 2018. No financial incentive was offered to participants.

To be included in the final sample, at minimum participants needed to have answered socio-cultural demographic questions, and whether or not they had undertaken fertility preservation. In total 442 people commenced the questionnaire. Of those, 28 people started the questionnaire but did not respond to any items. An additional five people started the questionnaire but did not complete the minimum questions required. Of the final sample of 409 participants, participants on average spent 12 min completing the questionnaire.

### Questionnaire

Participants were invited to complete an online questionnaire hosted on SurveyMonkey. No identifying information (i.e., names or email addresses) was collected as part of the questionnaire, and the IP addresses of participants were not tracked. SurveyMonkey servers use secure encryption, and access to the data collected is available only to the researchers (i.e., it is not available to SurveyMonkey staff). The first page of the questionnaire included background information to the research and links to a full information sheet and a list of support resources. Participants were asked whether they consented to participate in the questionnaire by selecting ‘Yes’ or ‘No’ after reading the information on the first page of the questionnaire. Participants selecting ‘Yes’ were directed to the start of the questionnaire. Those selecting ‘No’ were redirected to a page welcoming them to return and complete the questionnaire at another time.

The questionnaire, designed by the authors, drew upon previous research to focus on key variables which are likely to shape decision making around fertility preservation (e.g., [11]). The questionnaire first asked participants to provide demographic information, including gender, age, Australian State or Territory of residence, and sexual orientation. Participants were then asked to give information relating to their current relationship status, whether or not they had children (and/or planned to have children in the future), and if they already had children, whether they did so before or after transitioning gender. Participants were then asked ‘is it important to you that you share a genetic relationship with your children?’, ‘have you undergone gender affirming treatment which may impact on fertility?’ (and if so, what form this took), and ‘have you previously undertaken fertility preservation?’. Depending on participant responses to the last question, they were then directed to one of two pages.

Participants who indicated they had undertaken fertility preservation were asked an open-ended question about how they had made this decision, whether or not they had received advice or counselling prior to their decision, whether they had delayed their gender transition in order to undertake fertility preservation, and how likely they were to use stored gametes in the future (1 = not very likely, 2 = somewhat likely, 3 = quite likely, 4 = very likely). Participants who indicated they had not undertaken fertility preservation were asked an open-ended question about how they made this decision, whether they had received any advice or counselling prior to making this decision, and whether they would have considered delaying their gender transition in order to undertake fertility preservation. Both of these pages concluded by asking whether participants thought that fertility preservation should be available to all transgender and non-binary people. In sum, participants who indicated they had undertaken fertility preservation were presented with a total of 15 questions to answer, and participants who indicated they had not undertaken fertility preservation were presented with 14 questions to answer.

### Data analysis

Questionnaire data were exported from SurveyMonkey into SPSS 21.0. Descriptive statistics were generated for socio-cultural demographics (gender, Australian State or Territory, sexuality, and current relationship status, see Table 1 below), and for child-related demographics (have children or not, when they had children, and whether or not sharing a genetic relationship with children is important, see Table 2 below). Descriptive statistics were also generated for mean age at which fertility preservation was undertaken and likelihood of using stored gametes (see below), and treatments that may impact on fertility preservation and willingness to delay transition in order to undertake fertility preservation (see Table 4). Finally in terms of descriptive statistics, these were generated for whether or not participants had undertaken fertility preservation (and if so, what type), and whether or not they had received counselling or advice prior to their decision (see Table 5).

**Table 1:** Socio-cultural demographics (*N* = 409)

	Category	*N* (%)
Gender	Female	97 (23.7)
	Male	131 (32.0)
	Non-binary	149 (36.4)
	Agender	32 (7.8)
State or Territory	Australian Capital Territory	10 (2.4)
	New South Wales	92 (22.5)
	Northern Territory	6 (1.5)
	Queensland	102 (24.9)
	South Australia	50 (12.2)
	Tasmania	24 (5.9)
	Victoria	106 (25.9)
	Western Australia	19 (4.6)
Sexuality	Heterosexual	31 (7.6)
	Bisexual	65 (15.9)
	Gay	31 (7.6)
	Lesbian	37 (9.0)
	Pansexual	99 (24.2)
	Queer	109 (26.7)
	Asexual	37 (9.0)
Currently in Relationship	Yes	214 (52.3)
	No	195 (47.7)

**Table 2:** Child-related demographics

	Category	*N* (%)
Already have Children (*n* = 64)	Yes, and would like to have more	12 (18.8)
	Yes, and do not plan to have more	39 (60.9)
	Yes, and undecided about having more	13 (20.3)
When had Children	Before transitioning	48 (75.0)
	After transitioning	10 (15.62)
	Both before and after transitioning	6 (9.38)
Do not have Children (*n* = 345)	No, but would like to in the future	114 (33.0)
	No, and do not plan to have children	119 (34.5)
	No, and undecided about having children in the future	112 (32.5)

Drawing on previous research, inferential statistics were then performed on specific variables likely to be related to one another. These were: 1) having children already (or not) and gender, 2) views on the importance of genetic relatedness and fertility preservation, 3) age and gender-affirming treatments, 4) gender and having undertaken fertility preservation, 5) gender and willingness to delay gender transition in order to undertake fertility preservation, and 6) gender and the wish that fertility preservation had been undertaken. Additional tests were run on any relationship between advice or counselling received about fertility preservation and gender. Log-likelihood ratio tests (LLR) were used for all of the inferential statistics involving two categorical variables, given the existence of small cell sizes. For each LLR percentages are reported for each category so as to provide the reader with a breakdown of differences between groups. For the analysis of variance, Levene’s Test of Equality of Variance was used to test the assumption of equal variances, and to test the linearity of the data the Lack of Fit test was used. For each, results were non-significant, indicating that there were equal variances across groups examined, and that the data were linear.

Open-ended responses were analysed via a conventional content analysis, following the guidelines outlined by Hsieh and Shannon [20]. This involved 1) repeated readings of the data corpus, 2) developing codes by highlighting key words that capture frequently occurring concepts, 3) reducing codes in order to minimise overlaps, 4) examining codes for patterned responses, in order to group codes into categories, and 5) examining categories to determine whether or not they accurately reflect the data corpus. As noted by Hsieh and Shannon, a limitation of conventional content analysis is that it does not utilise member checking or inter-rater reliability. Given the questionnaire was anonymous, member checking was moot for the present study. In terms of inter-rater reliability, Hsieh and Shannon note that all analyses are subjective, and thus should be viewed as offering one interpretation derived by the researcher. Nonetheless, the content analysis undertaken by the first author was reviewed and confirmed by the second author.

## Results

### Participant demographics

Participants ranged in age from 18 to 72 years (*M* = 28.54, *SD* = 11.25). Other socio-cultural demographics are outlined in Table 1.

In terms of having children, 64 of the participants were already parents, and 345 participants were not (see Table 2). Of the parent participants, 64% became parents via their partner giving birth, 28% gave birth to their child, 5% were step parents, and 3% were foster parents. Further in terms of parent participants, 48% were female, 28% were non-binary, 18% were male, and 6% were agender, *LLR* (2, 64) = 53.97, *P* < 0.001. For participants who were not parents, 33% desired to have children in the future, of whom 44% were non-binary 36% were male, 12% were female, and 8% were agender, *LLR* (20, 397) = 47.87, *P* < 0.001.

### Importance of genetic relatedness

In terms of views on the importance of sharing a genetic relationship with children, 244 participants responded to this question, of whom 82 (33.6%) thought that genetic relatedness was important, and 162 (66.4%) thought it was not important. Focusing specifically on fertility preservation, of those who had undertaken fertility preservation 71% thought that genetic relatedness was important, and for those who had not undertaken fertility preservation 31% thought that genetic relatedness was important, *LLR* (1, 244) = 15.574, *P* = 0.008.

With regard to the open-ended question about the importance of genetic relatedness, a content analysis of the 91 responses to this question identified six key categories, as outlined in Table 3. Three categories pertained to participants who responded that genetic relatedness was *not* important to them. The most common response of these three categories was that participants were planning to adopt or foster, and thus had no interest in genetic relatedness. The other three categories pertained to participants who responded that genetic relatedness *was* important to them. The most common response of these three categories was a pragmatic acceptance that genetic relatedness was not possible, even if desired.

**Table 3:** Content analysis of importance of genetic relatedness

Viewed genetic relatedness as important	Category	*N*	Examples
No	Plan to adopt or foster children	21	“It doesn’t matter, I’m hoping to adopt”. “I feel it is selfish to prioritise a genetic relationship when there are so many children in the world without families”
No	A genetic relationship is not important	14	“Never saw why it made any difference”. “It’s not necessary for a child to be genetically related to their parents to have a normal and loving home environment”.
No	Perception of having ‘bad genes’	10	“I don’t want to pass on genetic mental illness”. “I have terrible genes and do not want them to continue”.
Yes	Pragmatic acceptance that genetic relatedness is not possible	19	“It is important to me, but ultimately not likely to be possible so I’m working on letting that go”. “I understood as I came to terms with my gender that I’d never bear my own children”.
Yes	Would like the option of having a genetic relationship	13	“I would like the option to have biological children”. “I want at least one other biological child for personal reasons, but it’s not actually important to me otherwise”.
Yes	Fine either way	14	“Ideally I would like to have another genetic child but I would also be happy to help raise a non genetic child”. “Both me and my partner would like children to share our genetics but if that’s not possible we want to adopt”.

### Gender-related medical treatment and its relationship to fertility

Participants were asked about any gender-affirming medical treatment they had undertaken which may impact on fertility. Responses are outlined in Table 4. A one-way between groups ANOVA was conducted to determine whether type of treatment undertaken differed by age. A statistically significant difference emerged, *F* (3, 237) = 14.49, *P* < 0.001. Post hoc comparisons using the Bonferroni test indicated that the mean age for participants who had undertaken gender affirming surgery was higher (*M* = 42.38, *SD* = 14.27) than the mean ages for puberty blockers (*M* = 20.50, *SD* = 3.20), current use of hormones (*M* = 29.43, *SD* = 10.93), and past use of hormones (*M* = 33.91, *SD* = 8.74). Also outlined in Table 4 are responses to questions about whether participants considered, or would have considered, delaying transition in order to preserve fertility. ‘Not applicable’ responses pertained primarily to non-binary people, who did not plan to undertake any medical treatments that would impact upon their fertility.

**Table 4:** Treatment and transition impact on fertility

	Category	*N* (%)
Treatment that may impact on fertility (*n* = 242)	Puberty blockers	6 (2.5)
	Hormones (current)	190 (78.5)
	Hormones (previously)	11 (4.5)
	Surgery related to reproductive organs	35 (14.5)
Delayed transition to preserve fertility (*n* = 26)	Yes	15 (57.7)
	No	11 (42.3)
Would have considered delaying transition to preserve fertility (*n* = 315)	Yes	64 (20.3)
	No	174 (55.2)
	Not applicable	77 (24.4)

### Fertility preservation decision making

Table 5 outlines rates at which fertility preservation was undertaken, the form this took, whether advice or counselling was given prior to decision making, and whether participants who had not undertaken fertility preservation now wished otherwise (and whether they had received advice or counselling prior to their decision not to undertake fertility preservation). The mean age at which participants had undertaken fertility preservation was 25.24 years (*SD* = 8.29). The mean for likelihood of using stored gametes was 2.28 (*SD* = 1.24), meaning that participants reported that they were only somewhat likely to use their stored gametes in the future. Of the 335 participants who responded to the question about whether or not fertility preservation should be offered to all transgender and non-binary people, 317 (94.6%) answered yes.

**Table 5:** Fertility preservation decision making

	Category	*N* (%)
Previously undertaken	Yes	28 (7.0)
fertility preservation (*n* = 398)	No	370 (93.0)
What form of fertility preservation (*n* = 28)	Stored gametes after beginning puberty but before starting hormones	19 (67.9)
	Stored gametes after commencing hormones	8 (28.6)
	Stored fertilized embryos	1 (3.6)
Given advice or counselling prior to fertility preservation (*n* = 28)	Yes	16 (57.1)
	No	12 (42.9)
Wish had undertaken fertility preservation (*n* = 318)	Yes	27 (8.5)
	No	120 (37.7)
	Unsure	44 (13.8)
	Was not available/offered	35 (11.0)
	Still an option	92 (28.9)
Given advice or counselling about future fertility preservation options (*n* = 308)	Yes	70 (22.7)
	No (and I am fine with that)	161 (52.3)
	No (and I wish I had)	49 (15.9)
	Not applicable	28 (9.1)

Of those who had undertaken fertility preservation, 53% were female, 21% were non-binary, 18% were male, and 8% were agender, *LLR* (2, 28) = 13.910, *P* < 0.001. In terms of delaying gender transition in order to have undertaken fertility preservation, of those who answered yes 66% were female, 20% were male, and 14% were non-binary, *LLR* (6, 26) = 14.806, *P* = 0.006. With regard to advice or counselling received prior to undertaking fertility preservation, there were no statistically significant differences in terms of gender. For those who had not undertaken fertility preservation, a majority (68%) indicated that they had not received any advice or counselling about fertility preservation. Of those who had not received advice or counselling, 39% were non-binary people, 30% were male, 20% were female, and 11% were agender, *LLR* (16, 318) = 77.625, *P* = 0.005.

In terms of decision making for the 28 participants who had undertaken fertility preservation, a content analysis of the 22 open ended responses indicated that participants primarily focused on who helped them make the decision, rather than why they made the decision per se*.* As reported in Table 6, the most common response was that participants made the decision to undertake fertility preservation due to their desire to have the option to have children in the future*.* Of the participants who had not undertaken fertility preservation, 155 responded to an open-ended question asking about how they had made this decision. A content analysis of these responses indicated six categories, as outlined in Table 6. The most common response was that cost was prohibitive to undertaking fertility preservation. Both having no interest in genetic relationships or having children, and the thought of children as inducing dysphoria, were also common responses.

**Table 6:** Content analysis of decision making about fertility preservation

Question	Theme	*N*	Examples
How did you make a decision to undertake fertility preservation?	Individual’s desire to have the option	11	“I decided to undertaken fertility preservation in case I still want to have children in the future” “I wanted to have children one day. I knew that soon my window would close and that my only option was to store sperm”
	Encouraged by medical professionals	4	“Recommended by health carers prior to starting HRT” “I started seeing an endocrinologist at a children’s hospital who brought up the prospect of fertility preservation”
	Encouraged by partner	3	“Wife wanted kids” “I was neutral on the issue and my partner felt strongly about having the option”
	Encouraged by family members	4	“My parents thought the procedure would be a good idea” “My mother is insistent on having grandchildren”
How did you make the decision not to undertake fertility preservation?	Cost was prohibitive	44	“It was not available to me due to the sheer cost of it” “I was not going to be able to afford it”
	Not interested in genetic relationships and/or children	35	“Don’t want genetic children, therefore fertility preservation is a non issue to me” “Never wanted children”
	Thought of children as dysphoria inducing	34	“I do not want children that are made through my genes. I do not want to bear children myself. The idea of childbirth adds to my dysphoria” “Conceiving and birthing a child sickens me and would humiliate me as a man”
	Have enough children already	20	“Nine children is enough” “I already have two children”
	Did not want to delay transition	11	“I wanted to begin my transition immediately as my priority was to treat the dysphoria. Future children were not a consideration” “Postponing hormones for possibly years until I was at the stage of my life to consider children was never an option”
	No genetic material available	11	“I can’t have children anymore due to a genetic disorder” “I had cancer and chemo early which meant that I had no material to store regardless of transition”

## Discussion

Drawing on a convenience sample of Australian transgender and non-binary people, this mixed-methods study found that only a minority of participants viewed being genetically related to children as important, though participants who had undertaken fertility preservation were more likely to hold this view. The relative lack of importance placed upon genetic relatedness amongst the sample echoes previous research with transgender (and in some studies non-binary) people in regards to fertility preservation and parenting [7–10], though differs significantly from research with cisgender populations, who have been found to strongly emphasise the importance of genetic relatedness [21].

Only a small number of participants had undertaken fertility preservation, and a majority of these participants had delayed their gender transition in order to preserve their fertility. For the majority who had not undertaken fertility preservation, delaying gender transition in order to preserve fertility was not considered. The relatively small number of participants who had undertaken fertility preservation also echoes previous research [13–15], as is the relative lack of willingness to consider delaying gender transition in order to preserve fertility [8, 11, 17]. Factors shaping the decision to undertake fertility preservation identified in the present research represent a novel contribution to the literature, whilst factors related to the decision not to undertake fertility preservation echo previous research [8, 9, 11, 17–19].

In terms of receiving advice or counselling about fertility preservation, over half of those who decided to preserve their fertility had received advice or counselling. For those who had not undertaken fertility preservation, less than a quarter had received advice or counselling. Previous research has paid little attention to whether advice or counselling is offered in the context of fertility preservation, despite the recommendations of the WPATH SOC that health care professionals discuss reproductive options prior to medical treatment. This finding is thus a novel contribution of the research reported in this paper.

### Study strengths and limitations

The strength of the present study is that it represents the largest study to date of fertility preservation decision making undertaken with a non-clinical sample of transgender and non-binary people. The mixed-methods approach allows for an understanding of both the rates at which fertility preservation occurred amongst the sample and aspects of decision making related to this, as well as something of the meaning that participants attributed to decision making. The inclusion of non-binary people in the present study adds an important dimension to previous studies that have focused primarily on transgender people. In terms of limitations, the participants were all Australian and were comprised of a convenience sample, meaning that the findings may not be generalizable to other contexts. Additionally, responses to the open-ended questions around decision-making were on average relatively brief, meaning that more in-depth analyses of this qualitative data were not possible.

### Directions for future research

Future research, including that planned as part of the present study, will benefit from focusing on experiences with fertility preservation. Whilst there are a small number of publications focused on this topic [8, 17, 22], a broader understanding is needed with regard to how diverse cohorts of transgender and non-binary people experience fertility preservation, as well as their thoughts on fertility and its relationship to gender more broadly. This may include research that explores in detail why many transgender and non-binary do not intend to have children, and how this relates to normative understandings of adulthood [23]. In other words, whilst it is important that researchers continue to investigate how transgender and non-binary people make decisions about fertility preservation, it is also important that researchers investigate how transgender and non-binary people choose to be child-free in the face of social norms about reproduction. In addition, future research could consider the experiences of transgender and non-binary people who undertake fertility preservation, including in terms of their desires to have children, and how they are impacted on by cisgenderism and norms around families and parenthood. Genetic reproduction and parenting, then, are but one part of the broader picture related to the decisions that transgender and non-binary people make about their lives.

## Conclusions

Given that the WPATH SOC [1] are currently under revision, the findings reported in this paper offer important insights. The current SOC include the suggestion that fertility preservation should be encouraged. The findings reported in this paper suggest a more cautious approach, one that most certainly involves raising the topic of fertility preservation and exploring all options, but which acknowledges that potentially for a majority of transgender people fertility preservation will not be of interest, and that for many non-binary people fertility preservation remains an option, depending on any medical treatments undertaken (and the barriers to these that may exist, as noted in the introduction to this paper). Importantly, however, for those in the present study who had not undertaken fertility preservation, the greatest number who had *not* received advice or counselling about this were non-binary people. This suggests the need for greater awareness about the importance of discussing fertility preservation options with non-binary people, both amongst clinicians who work with non-binary people, and non-binary people themselves. Finally, and given that the average age of participants in the present study who had undertaken fertility preservation was relatively young, this suggests that whether or not encouraging fertility preservation is useful will likely depend on the age of the person.

In terms of the wider clinical relevance of the findings, it is suggested that a focus on the meaning or importance of genetic relatedness may be a useful heuristic through which to provide counselling to transgender and non-binary people considering fertility preservation. This point may be especially salient given that those who had undertaken fertility preservation reported that they were on average only somewhat likely to use stored gametes in the future. Considering the high costs of fertility preservation including ongoing storage fees (alongside future costs of using the stored gametes), encouraging all transgender and non-binary people to undertake fertility preservation may not necessarily indicate best outcomes. Given the many pathways to parenthood available to transgender and non-binary people, other options besides genetic relatedness may be explored in order to mitigate the costs of fertility preservation if genetic relatedness is not viewed as important.

## Abbreviations

LGBT: Lesbian, gay, bisexual and transgender
SOC: Standards of Care
SPSS: Statistical Package for the Social Sciences
WPATH: World Professional Association for Transgender Health
